# Evaluation of a direct phage DNA detection-based Taqman qPCR methodology for quantification of phage and its application in rapid ultrasensitive identification of *Acinetobacter baumannii*

**DOI:** 10.1186/s12879-022-07493-1

**Published:** 2022-06-07

**Authors:** Jun Luo, Min Liu, Peng Wang, Qianyuan Li, Chunhua Luo, Hongping Wei, Yuanyuan Hu, Junping Yu

**Affiliations:** 1 The First College of Clinical Medical Science, China Three Gorges University, Yichang, China; 2 CAS Key Laboratory of Special Pathogens and Biosafety, Center for Emerging Infectious Diseases, Wuhan Institute of Virology, Chinese Academy of Sciences, Wuhan, 430071 Hubei China; 3 Central Laboratory, The First College of Clinical Medical Science, China Three Gorges University and Yichang Central People’s Hospital, Yichang, 443003 China; 4 Medical College, China Three Gorges University, Yichang, 443002 China; 5 Yichang Central People’s Hospital, Yichang, China

**Keywords:** Direct phage DNA detection-based Taqman qPCR, Quantification of phage, Viable *Acinetobacter baumannii*, Detection, Swab/bronchoalveolar lavage fluid

## Abstract

**Background:**

Rapid phage enumeration/quantitation and viable bacteria determination is critical for phage application and treatment of infectious patients caused by the pathogenic bacteria.

**Methods:**

In the current study, a direct phage DNA detection-based Taqman qPCR methodology for quantification of phage P53 and rapid ultrasensitive identification of *Acinetobacter baumannii* (*A. baumannii*) was evaluated.

**Results:**

The assay was capable of quantifying P53 phage DNA without DNA extraction and the detection limit of the assay was 550 PFU/mL. The agreement bias between the quantitative results of three different phage concentrations in this assay and double agar overlay plaque assay were under 3.38%. Through the built detection system, down to 1 log CFU/mL of viable *A. baumannii* can be detected within 4 h in *A. baumannii* spiked swab and bronchoalveolar lavage fluid samples. Compared with the Taqman qPCR that targets the conserved sequence of *A. baumannii*, the sensitivity of the assay built in this study could increase four orders of magnitude.

**Conclusions:**

The methodology offers a valid alternative for enumeration of freshly prepared phage solution and diagnosis of bacterial infection caused by *A. baumannii* or other bacterial infection in complicated samples through switching to phages against other bacteria. Furthermore, the assay could offer drug adjustment strategy timely owing to the detection of bacteria vitality.

## Introduction

Bacteriophage is a virus that can infect specific living host bacteria. Hundreds of progeny phages in a few minutes could be released from a single bacterial cell after a lytic phage infects host bacteria. Bacteriophages could be used for bacterial detection because of extraordinary specificity and high affinity [1–3]. Titers are of importance in phage application [4]. At present, the culture-based double agar overlay plaque assay is considered as a gold standard method for enumeration of bacteriophages and bacterial detection. However, the culture-based double agar overlay plaque assay is based on plaque counting including the steps of the isolation and enrichment of the bacteria ahead of time and a plaque assay, which is laborious and generally requires about 12–72 h of the incubation [5, 6]. Its efficiency is vulnerable to multiple factors such as medium used, bacterial host and plaque morphology.

New methods that are simple and stable are needed. Oriented layers of bacteriophages, bacteriophage based bioconjugates, detection of bacterial metabolites after phage lyses bacteria and other improved methods that used phage to detect bacteria have been developed [7–9]. Although most of these methods can shorten the detection time, the operation of many methods is relatively complex, and the ultimate detection depends on special precision instruments. Compared these methods mentioned above, quantitative PCR (qPCR) has been shown to be a valuable approach because of its characteristics of high-throughput, simplicity and rapidity [2, 10]. Phage-based qPCR, which could realize the rapid and specific identification of living bacteria, is of importance for the diagnosis of infected patients timely and determination of the efficacy of antibiotics. Reports of phage-based qPCR on the detection of pathogens are increasing because of some of the above characteristics and advantages. One of which, SYBR Green PCR is a common selection [11]. Recently, we reported a phage DNA detection-based Taqman qPCR for sensitive diagnosis of bloodstream infection, where a platform was built to detect *A. baumannii* (down to 10 CFU in 100 µL serum) in sera within 4 h without bacteria isolation and DNA extraction [12]. However, composition of different clinical sample types is complex, so the methodology should be evaluated in more sample types. Otherwise, the value of phage DNA detection-based Taqman qPCR in the enumeration/quantification of bacteriophage is neglected.

**Fig. 1:**
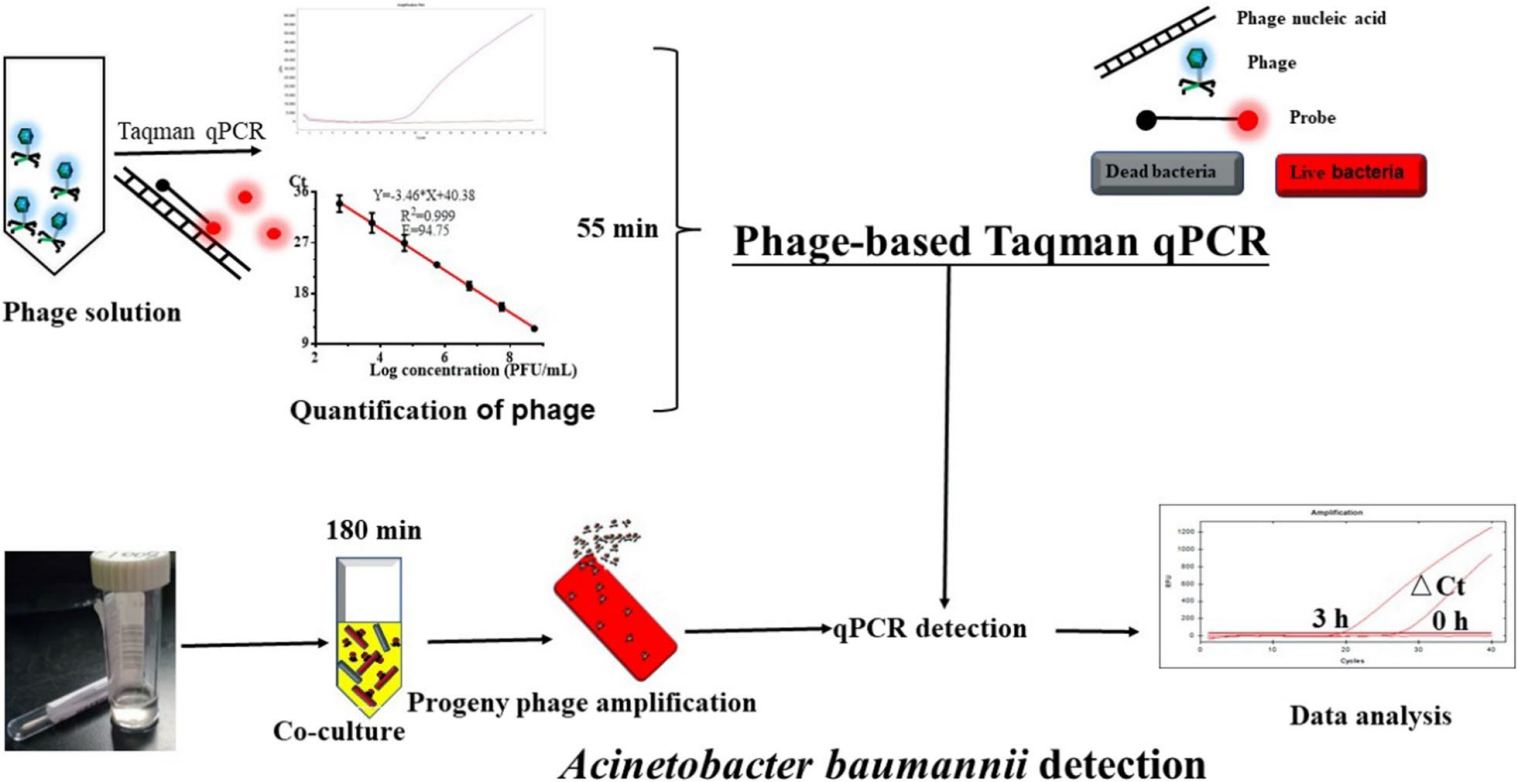
Schematic of direct phage DNA detection-based Taqman qPCR methodology for quantification of phage and its application in identification of host bacteria (not to scale)

As depicted in Fig. 1, compared with our previous report [12], more information/innovation could be found from the assay in the current study. Firstly, the accuracy and stability of the assay on the enumeration of freshly prepared phage solution is evaluated. Then, the spiked swab/bronchoalveolar lavage fluid samples used in this study are more complex than bloodstream. In addition, we enlarged the co-culture volume of phage and bacteria, improving the lower detection limit further. In this study, the system is applied to the noninvasive detection of viable *A. baumannii* in spiked swab/bronchoalveolar lavage fluid samples. The Ct changes between the time point 0 h and 3 h indicated whether there were *A. baumannii* or not in the specimen. Through adjusting the culture volume of the phage and bacteria to 1 mL, the detection limit for the bacteria in swab/bronchoalveolar lavage fluid samples could achieve 1 log CFU/mL. The sensitivity of the assay built in this study could increase four orders of magnitude, compared with the Taqman qPCR that targets the conserved gene of *A. baumannii*. The whole assay can complete within 4 h. All in all, a direct phage DNA detection-based Taqman qPCR methodology for quantification of phage and rapid ultrasensitive identification of host bacteria (*A.baumannii*) from spiked swab and bronchoalveolar lavage fluid samples is evaluated.

## Materials and methods

### Bacterial strains and culture condition

An *A. baumannii* strain, named LB8, isolated from a clinical pulmonary effusion sample, was used as an indicator strain in this study. The culture and preparation of bacteria are similar to the previously reported methods [12], LB8 preserved in 25% glycerol at 80 ºC was routinely streaked onto a Luria Bertani (LB, 10 g of NaCl, 5 g of yeast extract, 10 g of tryptone, and 15 g of agar per liter, where yeast extract, tryptone and agar were subscribed from Oxoid Ltd, Basingstoke, Hampshire, England) plate and incubated overnight at 37 °C. After picking a single colony on the plate into 5 mL fresh LB broth, the bacteria were grown overnight at 37 °C and 200 rpm. The bacterial suspension was centrifuged at 6000 rpm for 5 min. After discarding the supernatant, the bacterial pellet was re-suspended in 1 mL fresh LB broth. The OD_600_ of the bacteria solutions was adjusted to 0.45, which is corresponding to about 6 × 10^8^ CFU/mL of the bacteria (calculated by plate dilution). After the determination of the bacteria CFU, serial dilutions of the bacteria were adopted for the experiments.

### Preparation of spiked clinical swab and bronchoalveolar lavage fluid samples

*A. baumannii* spiked swab samples were prepared by delivering dilution series of 0.1 mL bacterial suspension directly onto skin swabs (the skin swabs were collected from college student volunteers and the skin swabs were also investigated by culture and plaque assays for the absence of either LB8 or p53). After resting for 10 min, the swab was stirred in a PCR tube containing 1 mL of physiological saline for 15 s. Spiked bronchoalveolar fluid samples were prepared by delivering dilution series of 0.1 mL bacterial suspension directly into 900 μL bronchoalveolar fluid that was collected from patients with lung infection (the bronchoalveolar fluid was investigated by culture and plaque assays for the absence of either *A baumannii* or p53).

### Preparation of the phage p53 solution

The preparation of bacteria is similar to the previously reported methods [12]. Briefly, the phage p53 against the host *A. baumannii* strain, LB8, was isolated from a sewage sample in the lab. The sequence of p53 was determined in the lab previously [12]. The preparation of phages p53 and determination of phage titers are based on our previous publication [12]. Briefly, the purification and PFU determination of the phage were realized by double-layer plate techniques. The mixture of 500 μL of host bacteria LB8(OD_600_ 0.8–1.2), 100 μL of phage p53 and 4 mL 0.7% water agar was poured onto LB agar plates and incubated for 12 h at 37 °C, the soft-agar layer was collected in 5 mL phage buffer made of 50 mM Tris–HCl (Tris base was bought from Sigma-Aldrich, Co, St. Louis, MO, USA) pH 7.5, 150 mM NaCl, 10 mM MgCl_2_, 2 mMCaCl_2_. After filtered by 0.22 μm sterile filter, the phage solution was obtained. Single clear plaques of a serial dilution of the phage solution were used for determination the concentration of a phage solution in PFU/mL. The PFU-determined phage solution was stored at 4 °C for further use.

### qPCR assay

The primers and probes were designed using Beacon Designer™ (version 8.13; www.premierbiosoft.com/molecular_beacons; Premier Biosoft International, Palo Alto, CA, USA) for specifically detecting p53 and *A. baumannii*, as listed in Table 1. The qPCR mixture had a final volume of 20 µL containing 10 µL of Premix Ex Taq™ (Probe qPCR) (product code: RR390A, Takara, Dalian, China), 0.4 µL of primers and probe (20 µM), 6.8 µL of distilled ddH2O and 2 µL of the DNA template prepared in the above. Dnase-free water was used as a negative control. The qPCR was run under the conditions as following: an initial denaturation step at 95 °C for 30 s followed by 40 cycles of denaturation at 95 °C for 5 s, annealing at 60 °C for 1 min with fluorescence acquisition on a AGS 4800 PCR instrument (Hangzhou AGS BioTech Co., Ltd., China).

**Table 1:** The sequences of the primers and the probes in this study

Target		Sequence (5ʹ–3ʹ)
P53	Forward	CGGATGTGGCAATATTAC
	Reverse	TTCCCATTTGCGATTTTG
	Probe	FAM- ATTCGATGTGGCACACCTGC-BHQ1
gltA	Forward	CGCATTATCTGCTTTCTA
	Reverse	TGGCTGACCTACAGTATA
	Probe	FAM- CACAACAACCTTGACATTGAAGACATC-BHQ1

### Evaluation of the efficiency and limit of detection of the Taqman qPCR on phage determination

The phages were serially diluted from 8 log PFU/mL to 2 log PFU/mL. Diluted phage samples were divided into two parts. One part was counted by double agar overlay plaque assay. The other part was detected by the direct phage-based Taqman qPCR. Meanwhile, three different phage solution including high titer, medium titer and lower titer were also detected based on above two methods.

### Determining the burst time of phage p53 in different matrix

To know whether different matrices would affect the phage detection using the phage DNA detection-based Taqman qPCR, ten-fold serially diluted phage solution ranging from 6 log PFU/mL to 3 log PFU/mL with in LB liquid medium, swab solution and bronchoalveolar fluid were detected by the assay. To determine the burst time of phage p53 in different matrix, 500 µL spiked clinical swab and bronchoalveolar lavage fluid samples as well as LB liquid medium, which contain 6 log CFU/mL bacterial suspension, were mixed with 500 µL p53 (3 log PFU/mL). The procedure of determining the burst time of phage p53 in different matrix are similar to the previously reported method [12]. Briefly, the mixture of the bacteria and the phage was co-incubated at 37 °C with shaking at 200 rpm. Lysis of the bacterial cells, as a result of phage replication, was monitored by the direct phage-based Taqman qPCR through taking 20 µL from the mixture every 10 min within initial 1 h, every 20 min within 2 h and every 30 min within 3 h. All the samples were stored at − 20 ºC immediately and used for the following the direct phage-based Taqman qPCR detection. The experiment was repeated in triplicate, and the mean burst time for LB8 was determined from the inflection point of the curves of the Ct changes vs. the initial incubation time.

### Optimization of phage concentration and the incubation time of p53 with LB8 for the detection of *A. baumannii*

Optimization of phage concentration and the incubation time of p53 with LB8 for the detection of *A. baumannii* is based on the previous method [12]. Through serially diluting the bacteria, different concentrations of LB8 (10^0^, 10^1^, 10^2^, 10^3^, 10^4^, 10^5^, and 10^6^ CFU/mL) in bronchoalveolar fluid were obtained according to the above description. Five hundred microliters LB8 in bronchoalveolar fluid broth were mixed with 500 µL of p53 at the concentrations of 10^2^, 10^3^, 10^4^, 10^5^, 10^6^ PFU/mL, respectively. Fifty microliters of the mixture of LB8 and p53 was immediately taken out and stored at − 20 °C, which was set as 0 min. Next, the mixture of LB8 and p53 were incubated at 37 °C with shaking at 200 rpm. Then 50 µL of the mixture was taken out every 40 min. All the taken out samples were stored at − 20 °C immediately for the direct phage-based Taqman qPCR detection of p53 after sampling was completed.

### Comparison of the sensitivities of methods using a Taqman qPCR assay based on the conserved sequence of bacteria and phage for the detection of *A. baumannii*

Five hundred microliters bacteria (ranging from 10^0^ to 10^6^ CFU/mL) in bronchoalveolar fluid mixed with 500 µL 10^3^ to 10^4^ PFU/mL p53 in phage buffer was co-incubated at 37 °C with shaking at 200 rpm. Fifty microliters of the mixture was taken out for Taqman qPCR detection of p53 at 0 and 180 min, respectively. Five hundred microliters bacteria (ranging from 10^0^ to 10^9^ CFU/mL) in bronchoalveolar fluid was also detected based on  a Taqman qPCR that targets gltA of *A. baumannii*. The experiments have been run for three times.

### Assay performance with spiked clinical swab/bronchoalveolar fluid samples

Spiked clinical swab and bronchoalveolar lavage fluid samples were prepared based on the above description. The ultimate bacterial suspension in spiked samples were 9 log CFU/mL, 6 log CFU/mL, 3 log CFU/mL and 1 log CFU/mL. The detection of phage and gltA of *A. baumannii* were based on the description of 2.8 in the material and method section.

### Taqman qPCR data analysis

Taqman qPCR data analysis has been illustrated in the previous report [12]. Briefly, there are two points should be stated when the sample contains *A. baumannii*. First, the Ct value at a time point should be smaller than the Ct value at the culture time 0 min, indicating that the phage is amplified at that time point and the sample might contain *A. baumannii*. Second, in consideration that the acceptable Ct value deviation of qPCR detection is normally lower than 0.5, 0.5 is a threshold for the change of Ct. If the Ct changes (ΔCt) between the initial and the other time point of the sample is above 0.5, the sample contains *A. baumannii*. Otherwise, there was no *A. baumannii* in the sample.

## Results

### Evaluation of the efficiency and limit of detection of the Taqman qPCR on phage determination

**Fig. 2:**
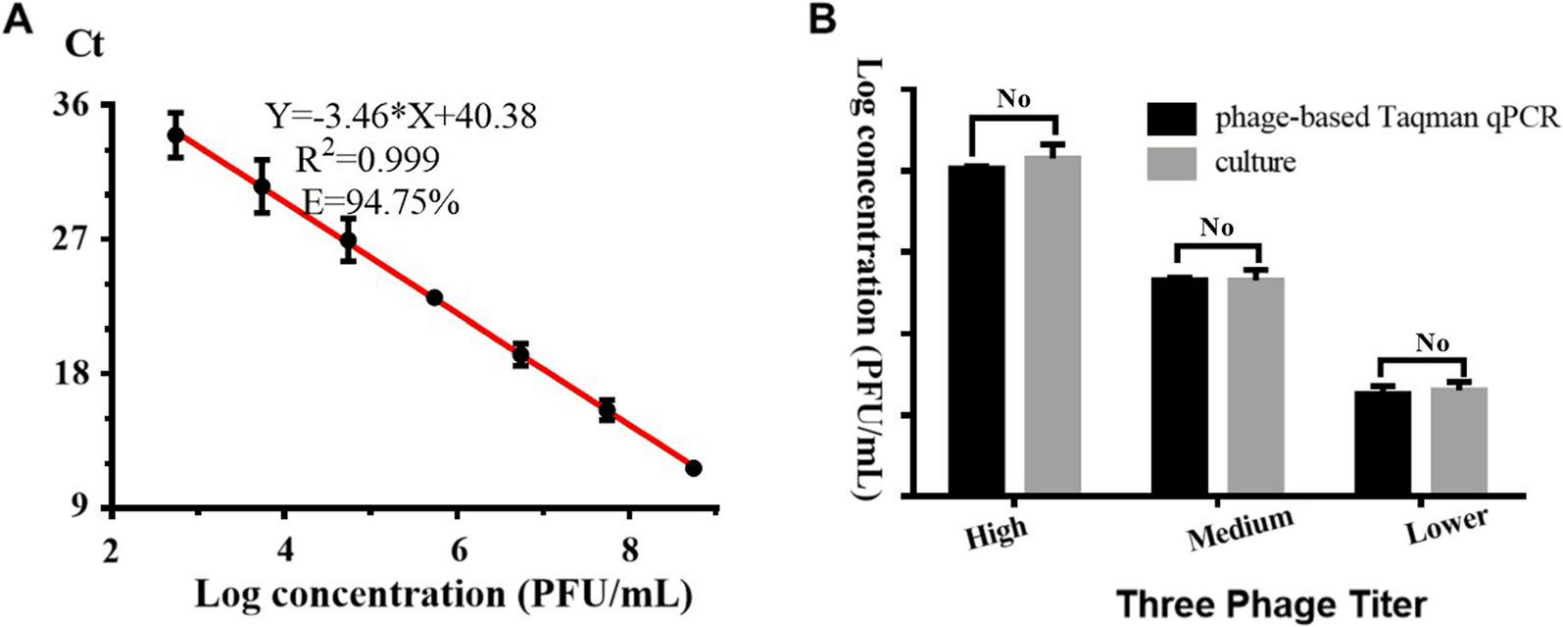
Efficiency of the amplification obtained after directly loading phages as the template without DNA extraction based on ten-fold serially diluting by the phage DNA detection-based Taqman qPCR (**A**) and quantification result comparison of three fresh phage solution between the phage DNA detection-based Taqman qPCR and double layer plaque assay (**B**). “No” indicates no statistical difference.

Phage solution was ten-fold serially diluted. The consistent detection was achieved from 2 Log concentration to 8 Log concentration, covering seven orders of magnitude. As shown in Fig. 2A, the amplification efficiency and correlation coefficient for p53 based on Taqman qPCR assay were 94.75% and 0.999. The detection limit of phage using Taqman qPCR was 550 PFU/mL. The agreement bias between the quantitative results of three different phage concentrations in this assay and double agar overlay plaque assay were under 3.38% and no statistical difference is observed (shown in Fig. 2B).

### Determining the burst time of phage p53 in different matrix

**Fig. 3:**
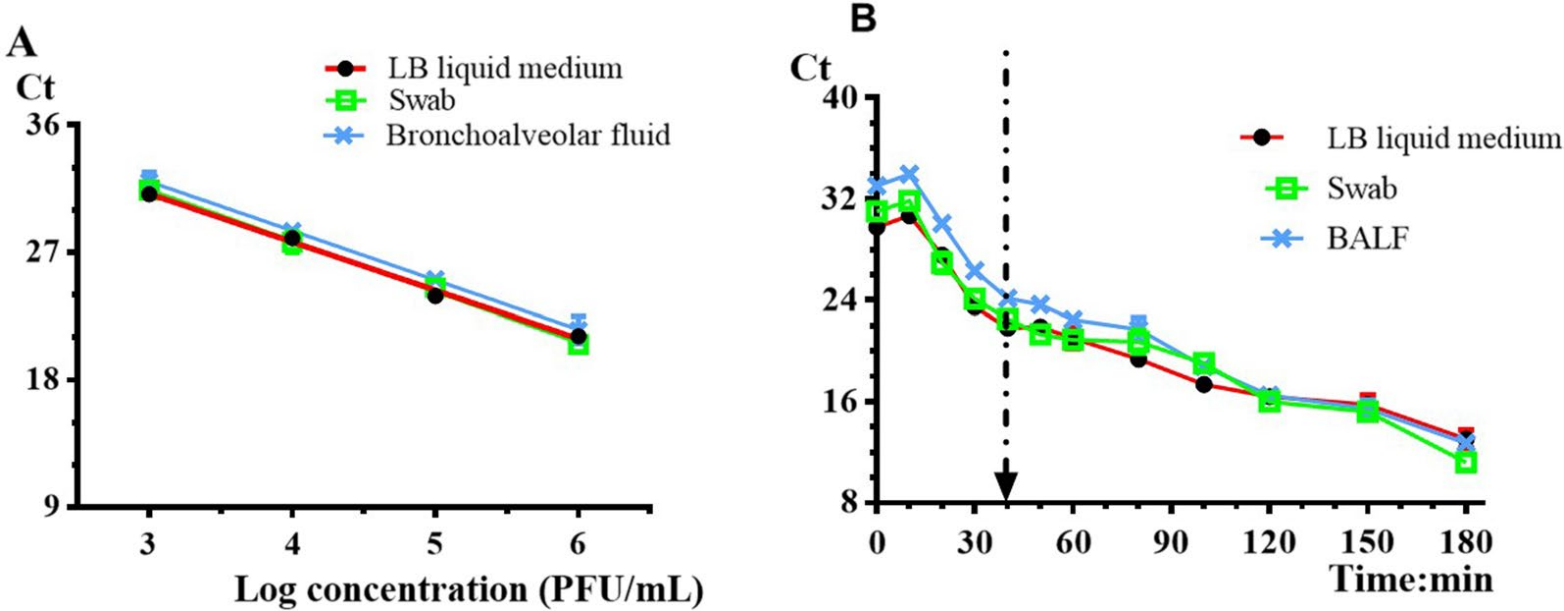
Efficiency of the amplification obtained from three different matrices using the phage DNA detection-based Taqman qPCR (**A**) and the kinetic curves of phage p53 in different matrix (**B**). The arrows indicate the culture time of 40 min, which is a short platform after a rapid phage amplification during the phage burst period and is used as the time interval for the following p53-based *A.baumannii* detection.

As shown in Fig. 3A, the amplification efficiency and correlation coefficient in LB liquid medium, swab solution and bronchoalveolar fluid were 94.7%/− 1.0, 90.3%/− 0.998 and 84.1%/− 0.992, respectively. To further know whether different matrices would affect the phage amplification and get the interval time for the detection of the host bacteria based on p53, the burst time of p53 was determined. The phage p53 concentration of 10^3^ PFU/mL and the bacteria LB8 concentration of 10^6^ CFU/mL were used. Plots of Ct values of qPCR at a different time point (every 10 min in the initial 1 h, every 20 min between 1 and 2 h and every 30 min between 2 and 3 h) vs. time were illustrated in Fig. 3B. In LB liquid medium, swab solution and bronchoalveolar fluid, the kinetic curves of phage were almost the same. Progeny phage particles were continuously produced, resulting in a progressive decrease of the Ct value as time went by (Fig. 3B). For LB liquid medium, swab solution and bronchoalveolar fluid, the kinetic curves of phage LB8 showed a latent period of about 20 min and a burst period of 120 min. As shown in Fig. 3B, 40 min is a short platform after a rapid phage amplification during the phage burst period. The results in this study combined with our previous report also have illustrated that the phage amplification was able to be discriminated by the Taqman qPCR at about 40 min. Based on above description, 40 min is used as the time interval for the following p53-based *A. baumannii* detection, which could help us ensure the ultimate optimum detection time in one simple and rapid way.

### Optimization of phage concentration and incubation time of p53 with LB8 for the detection of *A. baumannii*

**Fig. 4:**
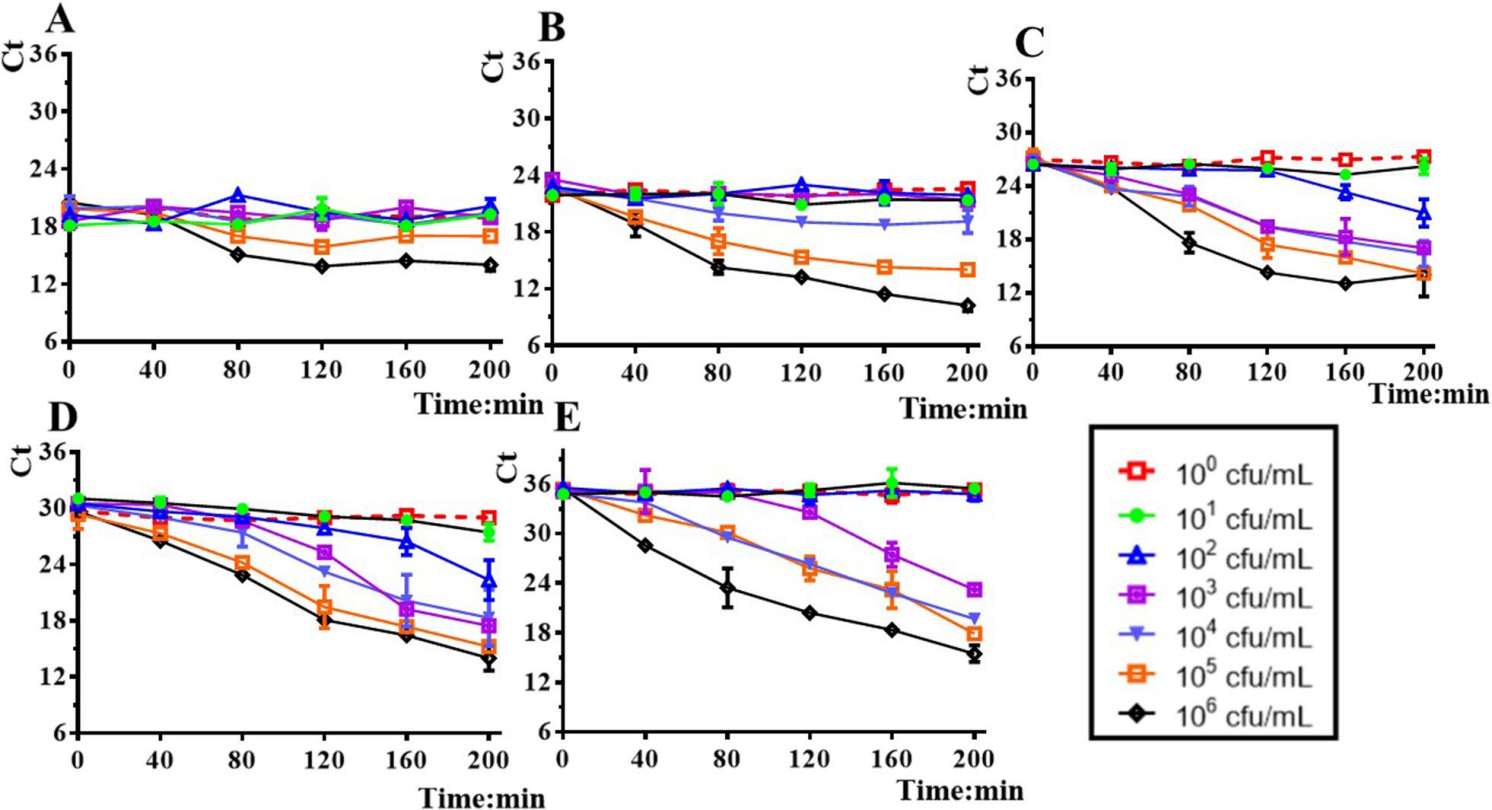
Optimize the phage concentration to detect different concentrations of the host *A. baumannii* LB8 (From 10^0^ CFU/mL to 10^6^ CFU/mL). The Ct values vs. the culture time of the phage p53 mixed with *A. baumannii* at the phage concentration of **A** 10^6^ PFU/mL, **B** 10^5^ PFU/mL, **C** 10^4^ PFU/mL, **D** 10^3^ PFU/mL, **E** 10^2^ PFU/mL. The errors were obtained from three independent experiments

Bronchoalveolar fluid was selected as a complex matrix for the optimization of phage concentration and incubation time. Through optimization of phage concentration, the sensitivity of the detection for the host bacteria can be improved. The optimization of the concentration of p53 was started from 10^2^ PFU/mL since the lowest concentration of 550 PFU/mL can be detected based on the method. The host concentrations, ranging from 10^0^ to 10^6^ CFU/mL, mixed with serial tenfold concentration of p53 (varying from 10^2^ to 10^6^ PFU/mL, the totally 5 concentrations of 10^2^, 10^3^, 10^4^, 10^5^, 10^6^) were detected. The samples were collected at 40 min intervals for the detection of qPCR until 200 min and the corresponding Ct values were recorded. The plots of Ct values vs. the culture time of the mixture of p53 and LB8 were illustrated in Fig. 4. When the concentration of p53 was higher than 10^4^ PFU/mL or at low concentration of p53 (10^2^ PFU/mL), decreasing detection limit of the bacteria can be observed as shown in Fig. 4A–C and 4E. As revealed in Fig. 4C, D for the phage p53 concentration of 10^3^ to 10^4^ PFU/mL, the sensitivity was the highest and the detected range of the bacteria is the broadest, where 10 CFU/mL of *A. baumannii* was detected at the time point from 160 to 200 min. Therefore a phage p53 concentration of 10^3^ to 10^4^ PFU/mL was selected for the following detection of bacteria. Though longer incubation time will improve the sensitivity of this assay (shown in Fig. 4C, D), the co-culture time of the phage and the bacteria was set as 180 min, considering the sensitivity and the rapidity of the detection.

### The detection limit comparison of *A. baumannii* using Taqman qPCR assay based on the conserved sequence of bacteria and phage

**Fig. 5:**
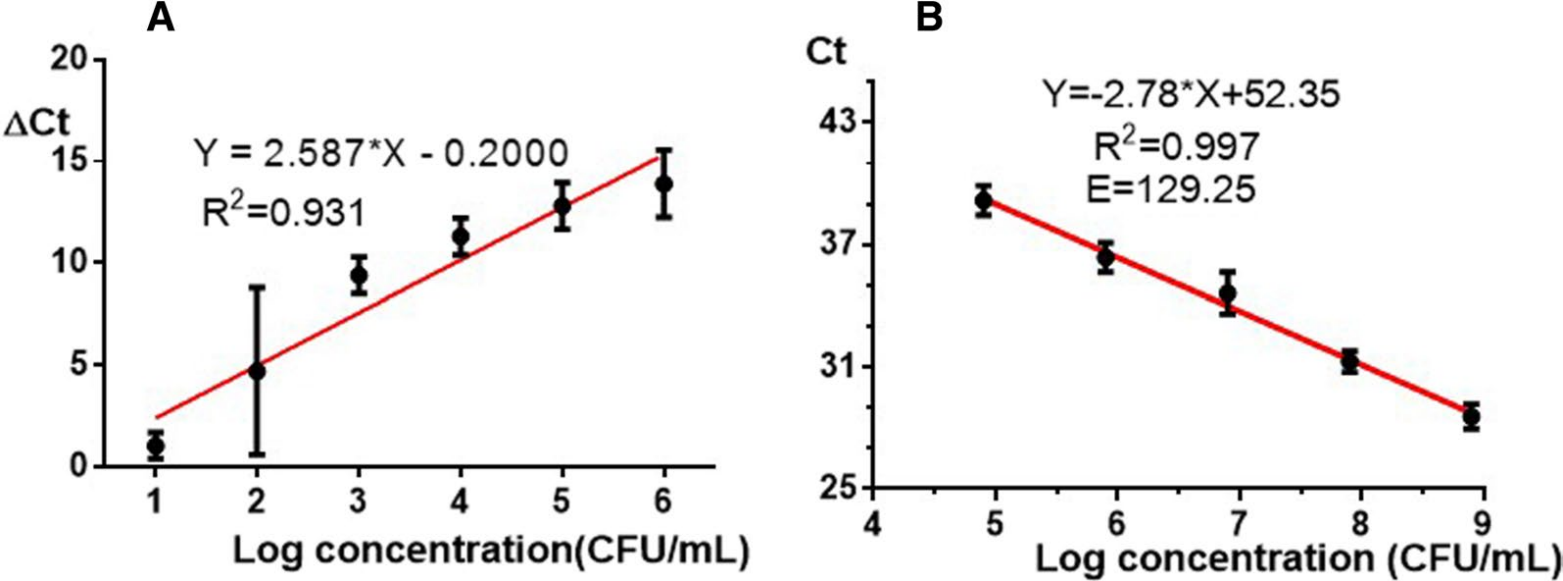
The detection limit comparison of *A. baumannii* using Taqman qPCR assay that was based on phage (**A**) and bacteria (**B**), respectively

Bronchoalveolar fluid was selected as the matrix to compare the detection limit for detecting *A. baumannii* using Taqman qPCR assay based on the conserved sequence of bacteria and phage. As shown in Fig. 5A, the detection limit of *A. baumannii* was 1 log CFU/mL using the phage DNA detection-based Taqman qPCR. The correlation coefficient is 0.931. Meanwhile, as revealed in Fig. 5B, the detection limit of *A. baumannii* was 5 log CFU/mL using the Taqman qPCR based on the conserved sequence of bacteria, and the amplification efficiency and correlation coefficient were 129.25% and 0.997.

### Assay performance in complex clinical samples

For spiked clinical samples in two complex matrices of swab and bronchoalveolar fluid samples, two Taqman qPCR assays were compared (Table 2). As mentioned above, the detection limit of Taqman qPCR assay based on gltA is 5 log CFU/mL. The detection limit could down to 3 log CFU/mL after 3 h of incubation. While the detection limit of the phage DNA detection-based Taqman qPCR for 3 h incubation could reach 1 log CFU/mL.

**Table 2:** Assay performance of phage DNA detection-based Taqman qPCR and gltA-based Taqman qPCR for spiked clinical swab and bronchoalveolar fluid samples

LB8		Taqman/gltA				Phage DNA detection-based Taqman qPCR		
Simulate samples		Swab		Bronchoalveolar fluid		Swab	Bronchoalveolar fluid	Phage
Time(h)		0	3	0	3	3	3	0
**9**		23.6 ± 0.5	22 ± 0.1	25.2 ± 0.6	22.5 ± 1.0	22	14.7 ± 0.4	31.2 ± 0.4
	△Ct					9.2	16.5	
**6**		32.7 ± 0.9	28.5 ± 0.3	30.3 ± 0.7	29.3 ± 0.6	28.1 ± 0.4	25.5 ± 1.2	
	△Ct					3.1	5.6	
**3**		U	36.1 ± 0.9	U	35.5 ± 1.5	26.5 ± 1.7	29.2 ± 0.1	
	△Ct					4.7	2.0	
**1**		U	U	U	U	29.8 ± 0.8	30.4 ± 1.2	
	△Ct					1.4	0.8	

Bold indicates 9, 6 ,3 and 1 mean that the initial bacteria amount in specimens is 9/6/3/1 log concentration(CFU/mL)

## Discussion

The application of bacteriophages in the identification and treatment of bacterial infection have become more and more extensive. Among them, the accurate quantification of the number of phages is critical for their applications. The agreement bias between the quantitative results of three different phage concentrations in this assay and double agar overlay plaque assay indicate that the quantification of phage solutions based on direct phage DNA detection-based Taqman qPCR is stable, fast and accurate, which is consistent with other studies [13, 14].

The sequence alignment results of primers and probe, used in the phage DNA detection-based Taqman qPCR, show that there is only one *Acinetobacter* phage with high coverage and high similarity to the amplified sequence of p53, indicating that amplification reaction is specific. In many previous reports, accurate quantification of phages by PCR required efficient removal of intact host bacterial cells from the samples, while the established method in this study could omit the process because of specific primers and probe. The assay in this study can be completed in 1 h, which shorten the accurate quantification time, and could realize the real-time use of bacteriophages when fresh phage solution is prepared.

*Acinetobacter baumannii* is a ubiquitous, Gram-negative, opportunistic pathogen, which is mainly responsible for hospital and community-acquired infections [15–17]. The increasing number of multidrug-resistant *A. baumannii* have become potential serious threat to public health. Since safe and effective therapeutic options in carbapenem-resistant *Acinetobacter baumannii* infections are severely limited, characterization of these isolates by phenotypic and molecular methods are important to provide information on the epidemiological characteristics of these pathogens [18, 19]. The genome analysis shows that *A. baumannii* contain mobile genetic elements associated with antimicrobial resistance genes (ARGs), which lead to that *A. baumannii* can develop drug resistance to more antimicrobial agent [20, 21]. Biofilm-forming capacity is also potent way for bacterial survival in the presence of antibiotics, which could increase the treatment difficulty of *A. baumannii* infection [22–24]. Reasonable antibiotic treatment can help patients treat effectively and reduce the risk of antibiotic resistance. The direct phage DNA detection-based Taqman qPCR assay in this study could offer a drug adjustment strategy timely owing to the detection of bacteria vitality to avoiding potential drug resistance. The assay in this study is based on detection the phage DNA copies change by the consideration of change in Ct (Δ Ct) instead of Ct value, which could remove the need to eliminate seed phages (by DNase treatment) or bacterial cells before detection. The assay can be completed in 4 h, which can satisfy the requirement of rapid diagnosis. By the current developed assay, 1 log CFU/mL of the host bacteria in spiked swab/bronchoalveolar fluid samples can be detected through phage amplification. Compared with the Taqman qPCR that targets the conserved gene of bacteria, the sensitivity of the assay built in this study could increase four orders of magnitude (shown in Fig. 5). Results in the Table 2 illustrated that even if the bacteria were enriched for 3 h in LB liquid medium, the sensitivity of the Taqman qPCR that targets the conserved gene of bacteria is still much lower than the method built in this study. Compared with our previous report [12], the results in this study prove that increasing the initial specimen volume can further improve the sensitivity of detection. Otherwise, increasing DNA template volume based on amplifying reaction would also improve the sensitivity of detection.

Taqman qPCR based on target bacterial DNA might produce false positive results sometimes when bacteria died but the nucleic acid has not totally degraded. The direct phage DNA detection-based Taqman qPCR assay could reduce this kind of false positive results because phages propagate only through viable host bacteria. Especially for patients taking medicine, the method allows the identification of live bacteria could help patients adjust the dosage in time. The method can also be applied for bacteria susceptibility detection because the amount of the viable bacteria will be different for drug-sensitive and drug-resistant bacteria when bacteria and antibiotics are co-incubated for 1 or 2 h. Otherwise, this method provides ideas for rapid and accurate phage enumeration when preparing fresh phage solution and monitoring phage amount change in phage therapy.

In the current study, there are still some questions to be solved in the future. Like many phage detection methods, the specificity of phages on host bacteria would restrict the application of detection methods, which might be solved by phage modification or phage cocktail in the future [25]. To evaluate the clinical application prospect of this method, this method needs to be verified by more real clinical samples in the future. In addition, the qPCR method is not able to discriminate between infective and defective virus particles, comparable to traditional culture-based double agar overlay plaque assay, while through the amplification of the burst phage after infection of bacteria, the developed method can reach ultrahigh sensitivity to detect live *A.baumannii*. During the detection of bacteria in clinical samples, a positive control with live bacteria should be set and we can ensure the viability of the phage and avoid false negative through the increase of phage DNA after incubation for the positive control.

## Conclusion

Here, a direct phage DNA detection-based Taqman qPCR methodology for quantification of phage P53 and its application in rapid ultrasensitive identification of *A. baumannii* has been evaluated. Through the amplification of the burst phage after infection of bacteria, the developed method can reach the real time quantification of phage and ultrahigh sensitivity to detect live *A. baumannii*. By the built detection system, down to 1 log CFU/mL of viable *A. baumannii* can be detected within 4 h in spiked swab and bronchoalveolar lavage fluid without any steps of bacteria isolation and DNA purification. However, to prove the method to be general to detect other bacteria, we should isolate various phages against different bacteria and evaluate the feasibility of this method in the detection of more live bacteria in the future. All in all, the developed assay will help to improve diagnosis of microbiological infections and treatment of these infectious patients.

## Data Availability

The data that support the findings of this study are available on request from the corresponding author. The data are not publicly available due to privacy or ethical restrictions. DNA sequencing data of p53 can be obtained from the website: https://pan.baidu.com/s/1a2hhqSwUN3zxd9FJaDGw9A, extraction code:0203.

## Abbreviation

*Acinetobacter baumannii*: *A. baumannii*
